# Pentraxin-3 as a poor marker of fibrosis in metabolic dysfunction-associated steatotic liver disease among older adults: findings from the PolSenior2 substudy

**DOI:** 10.3389/fmed.2025.1445973

**Published:** 2025-02-24

**Authors:** Aleksander Jerzy Owczarek, Joanna Musialik, Adrian Stefański, Małgorzata Mossakowska, Katarzyna Zięba, Andrzej Więcek, Jerzy Chudek, Magdalena Olszanecka-Glinianowicz

**Affiliations:** ^1^Health Promotion and Obesity Management Unit, Department of Pathophysiology, Faculty of Medical Sciences in Katowice, Medical University of Silesia in Katowice, Katowice, Poland; ^2^Department of Nephrology, Transplantology and Internal Medicine, Faculty of Medical Sciences in Katowice, Medical University of Silesia in Katowice, Katowice, Poland; ^3^Department of Preventive Medicine and Education, Faculty of Medical Science, Medical University of Gdańsk, Gdańsk, Poland; ^4^Study on Aging and Longevity, International Institute of Molecular and Cell Biology, Warsaw, Poland; ^5^Department of Internal Medicine and Oncological Chemotherapy, Faculty of Medical Sciences in Katowice, Medical University of Silesia in Katowice, Katowice, Poland

**Keywords:** PTX-3, FIB-4, MASLD, fibrosis, NASH, the elderly population

## Abstract

**Introduction:**

The study aimed to assess the relationship between plasma pentraxin 3 (PTX-3) levels and the potential diagnosis of fibrosis in metabolic dysfunction-associated steatohepatitis (MASH) in older adults. This was assessed using the Fibrosis-4 index (FIB-4), NAFLD fibrosis score (NFS), and Hepamet fibrosis score (HFS).

**Materials and methods:**

The subanalysis included 2,397 older adults (aged 60 years and older) from the population-based PolSenior2 study, all of whom had risk factors for metabolic dysfunction-associated steatotic liver disease (MASLD) and underwent PTX-3 assessment. The participants were divided into two subgroups according to the FIB-4 values (≤2.67 and > 2.67), three subgroups according to the NFS values (< −1.455, −1.455, and 0.675, and > 0.675), and three subgroups according to the HFS values (< 0.12, 0.12 and 0.47 and > 0.47).

**Results:**

The empirical cutoff points for PTX-3 levels as a potential marker of liver fibrosis were assessed separately for women and men. In women, the cutoff points for PTX-3 levels based on ROC curve analyses ranged from 1.96 to 2.30 ng/mL (an AUC ranging from 0.596 to 0.643, sensitivity between 39.1 and 61.7%, and specificity between 56.1 and 79.6%). In men, a significant cutoff point was established for FIB-4 (an AUC of 0.549, sensitivity of 39.4%, and specificity of 69.6%). Overall, the accuracy was poor.

**Conclusion:**

Our study suggests that plasma PTX-3 levels are not sensitive enough to be used as a non-specific marker of liver fibrosis in older adults.

## 1 Introduction

Non-alcoholic fatty liver disease (NAFLD), later referred to as metabolic dysfunction-associated fatty liver disease (MAFLD) and more recently as metabolic dysfunction-associated steatotic liver disease (MASLD), is the most common cause of chronic liver damage in developed countries. The spectrum of liver abnormalities in patients with MASLD is wide and includes steatosis, steatohepatitis, cirrhosis, and liver failure, and are associated with a high risk of hepatocellular carcinoma (1).

The ‘gold standard’ in diagnosing and determining the severity of fibrosis in patients with steatohepatitis is liver biopsy. However, this invasive test is associated with a risk of complications (2). More commonly, liver fibrosis in MASLD is diagnosed using ultrasound-based elastography (controlled attenuation parameter – CAP) and magnetic resonance-based elastography (3). However, these methods are expensive, and their availability in daily clinical practice is limited. Therefore, biochemical predictors of fibrosis severity in patients with MASLD are constantly being sought. Currently, there are several indicators of liver steatosis and fibrosis, some of them may be used in everyday clinical practice due to their easy availability, including FLI (4), LAP (5), ION (6), NAFLD-LFS (7), APRI (7), FIB-4 (8), NFS (9), and fibrometer NAFLD (10). Among the most frequently used factors considered in these indicators are age; gender; BMI; platelet count; ALT, AST, and GGT activities; triglyceride levels; and waist circumference (4–10).

Since the FIB-4 scores and NFSs are affected by patient age and body mass index and have limited accuracy in identifying patients with advanced fibrosis, the Hepamet fibrosis scoring system was recently developed. The HFS includes age, sex, diabetes, HOMA-IR, AST, albumin, and platelets. It should be noted that the HFS has higher accuracy than the FIB-4 scores and NFSs in identifying patients with advanced fibrosis, and it does not require adjusting for age (11). However, single biochemical markers are constantly being sought to determine the degree of liver fibrosis. It was reported that high-sensitivity C-reactive protein (hs-CRP) levels may have some clinical usefulness in the diagnosis of NASH (12). Furthermore, pentraxin 3 (PTX-3) was suggested as a marker of fibrosis in metabolic dysfunction-associated steatohepatitis (MASH, formerly known as NASH).

Pentraxins are a superfamily of acute-phase reactants. Their characteristic feature is a cyclic multimeric structure. The C-reactive protein (CRP) and serum amyloid P-component (SAP) are well-known short pentraxins, while pentraxin 3 (PTX-3) is the long one (13). In a middle-aged population, plasma PTX-3 levels above 2.45 ng/mL were found to effectively discriminate between patients with NASH and those without, with 91.1% sensitivity and 71.4% specificity. Furthermore, plasma PTX-3 levels correlated with the NFSs and histologically verified fibrosis stage and steatosis grade (14). In another study, it was found that plasma PTX-3 levels were significantly higher in NAFLD fibrosis stages 3–4 and correlated with them (15). In a population of adolescents with obesity, PTX-3 concentrations above 3.03 ng/mL were shown to distinguish between fatty liver and NASH, with 89% sensitivity and 86% specificity (16). In addition, a study of young Turkish adults showed that elevated plasma PTX3 levels were associated with the presence of fibrosis assessed based on liver biopsy in patients with NAFLD. This association was independent of metabolic syndrome components and other fibrosis predictors (17). Notably, PTX-3 levels were also linked to mortality in patients with liver cirrhosis. In this population, the Kaplan–Meier probability of survival was 77.0% in patients with PTX-3 < 5.3 ng mL and 53.5% with PTX3 > 5.3 ng/mL (18). On the contrary, another study found no correlation between plasma PTX-3 levels and histological steatosis, inflammation, or fibrosis stage in patients with hepatocellular carcinoma (19).

There are no studies exploring the relationship between plasma PTX-3 levels and liver fibrosis in older adults. Therefore, to assess this relationship in a large cohort of older adults, participants from a population-based Polsenior 2 study were assessed for plasma pentraxin 3 (PTX-3) levels and their potential association with fibrosis diagnosis in metabolic dysfunction-associated steatohepatitis (MASH), based on the FIB-4 scores, NFSs, and HFSs.

## 2 Materials and methods

### 2.1 Study design and setting

The PTX-3 substudy group included 2,397 (1,728 women and 1705 men) from the PolSenior2 study, all aged 60 years and older. These participants had available plasma samples, stored at −70°C, remaining after per-protocol assessments. The PolSenior2 study design and methodology are described elsewhere in detail (20).

Seven equal-sized age cohorts (60–64, 65–69, 70–74, 75–79, 80–84, 85–89, and 90 years and older) were recruited from the population using a three-stage, stratified, proportional draw, with a response rate of 56.9%. Trained nurses conducted three home visits per participant, where they performed face-to-face interviews (with a questionnaire survey), comprehensive geriatric assessment, and measurements of body mass, height, waist circumference, and blood pressure. Additionally, blood and urine samples were collected.

**Figure 1:**
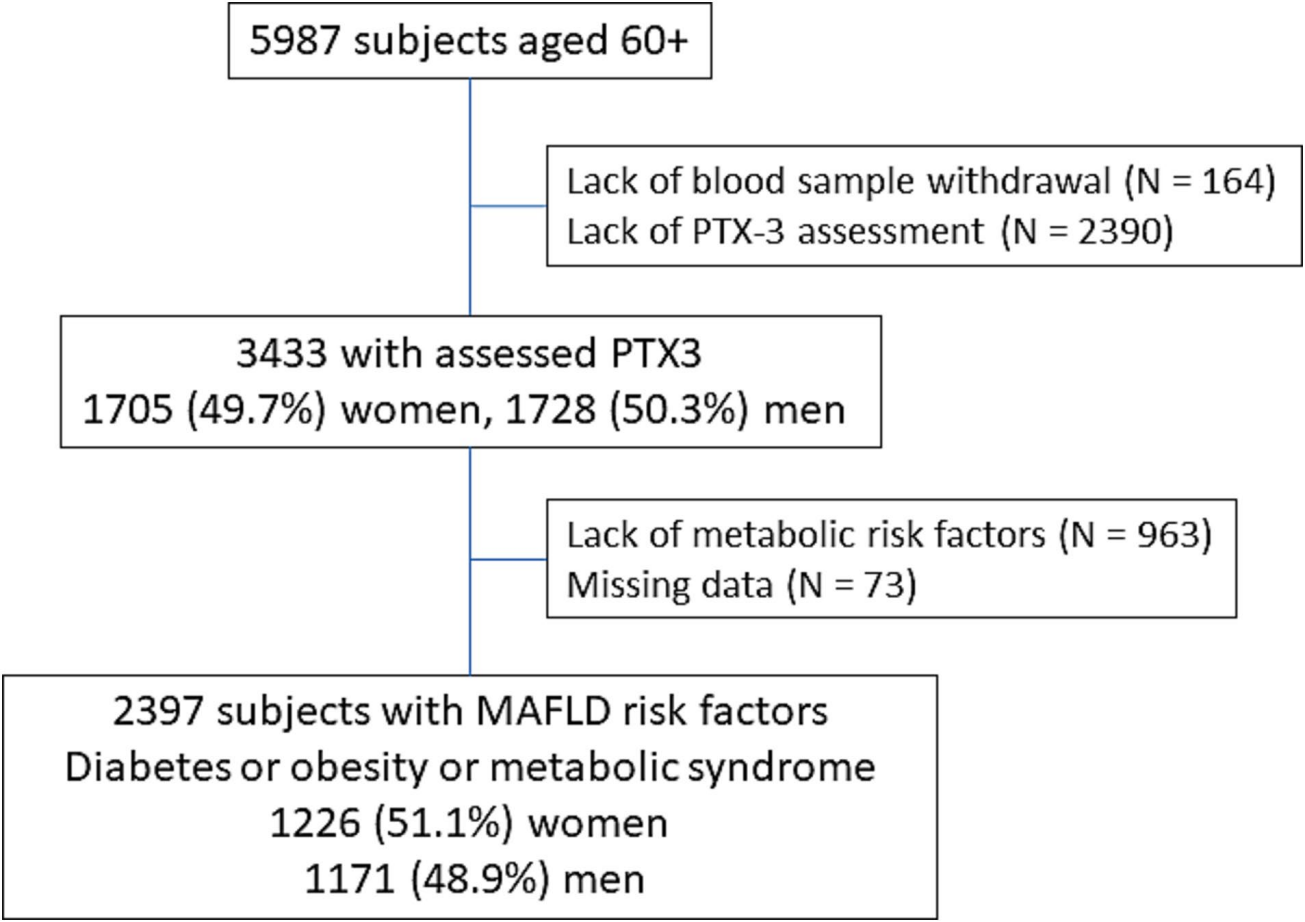
Flowchart of the analysis.

This analysis included Polsenior2 study participants with MASLD risk factors (diabetes, obesity, or metabolic syndrome) that were identified in 2,397 out of 3,433 PTX-3 substudy subjects (1,226 women and 1,171 men), as shown in the analysis flowchart (Figure 1).

### 2.2 Biochemical measurements

Blood morphology was assessed in local laboratories. Serum total cholesterol, LDL cholesterol, HDL cholesterol, triglycerides, bilirubin, glucose, insulin, albumin, CRP, and serum activity of ALT, AST, and GGT were assessed using automated systems [Siemens Healthcare kits as previously described (20)] in a single certified laboratory, with accuracies of 1.3, 1.6, 1.6, 2.5, 6.3, 1.1, 3.6, 2.4, 7.4, 2.8, 1.7, and 1.5%, respectively.

Plasma PTX3 levels were determined using the ELISA method at the Department of Pathophysiology, Faculty of Medical Sciences in Katowice, Medical University of Silesia in Katowice (BioVendor, Brno, The Czech Republic). The limit of quantification (LOQ) was 0.08 ng/mL, with intra- and inter-assay coefficients of variations of 6 and 12%, respectively.

### 2.3 Data analysis

Obesity was diagnosed according to the WHO criteria (21), and visceral obesity according to the International Diabetes Federation criteria (22).

Diabetes was diagnosed based on medical history, medication use, or fasting serum glucose ≥126 mg/dL. Insulin resistance in a group of non-diabetic subjects was determined based on the homeostasis model assessment of insulin resistance (HOMA-IR) method, calculated using the standard formula: HOMA-IR = fasting serum insulin (μIU/mL) x fasting glucose (mg/dL)/405.

In addition, FIB-4 (8) scores, NFSs (10), and HFSs (11) were calculated. The participants were divided into subgroups based on FIB-4 scores (≤ 2.67 and > 2.67) and into three subgroups based on NFSs (< −1.455, −1.455 to 0.675, and > 0.675). NFSs < −1.455 predict the absence of advanced fibrosis, scores between −1.455 and 0.675 are considered indeterminate, and scores >0.675 predict the presence of advanced fibrosis. The negative predictive value of no advanced fibrosis is estimated at 93%, while the positive predictive value of advanced fibrosis is at 90% (10). Moreover, based on the HFSs, the study group was divided into three subgroups (< 0.12, 0.12 and 0.47, and > 0.47). The HFSs <0.12 predict the absence of advanced fibrosis, scores between 0.12 and 0.47 are considered indeterminate, and scores >0.47 predict the presence of advanced fibrosis. The negative predictive value of no advanced fibrosis is estimated at 95.2%, while the positive predictive value of advanced fibrosis is estimated at 72.9% (11).

### 2.4 Statistical analysis

Statistical analyses were performed using STATISTICA 13.0 PL (TIBCO Software Inc., Palo Alto, CA, United States), and Stata SE 13.0 (StataCorp LP, TX, United States). Statistical significance was set at a *p*-value below 0.05. All tests were two-tailed. Imputations were not performed for missing data. Nominal and ordinal data were expressed as percentages. Interval data were expressed as the mean value ± standard deviation (SD) in the case of normal distribution. In the case of data with skewed or non-normal distribution, they were expressed as the median, with lower and upper quartiles. The distribution of variables was evaluated by the Anderson–Darling test and the quantile-quantile (Q–Q) plot. The homogeneity of variances was assessed by the Levene test. Nominal and ordinal data were compared with the χ^2^ test. Comparisons between groups for interval data were made with either the Student’s t-test in case of normal data distribution or the Mann–Whitney U-test otherwise or with a one-way analysis for more than two groups. The cutoff points for PTX-3 according to FIB-4 > 2.67 or the positive NFSs or HFSs >0.47 were calculated based on the ROC curve analysis. The chance for fibrosis based on the PTX-3 level was shown with odds ratio (OR) and corresponding 95% confidence interval (CI) and *p*-value.

## 3 Results

### 3.1 FIB-4

FIB-4 values >2.67, indicating advanced fibrosis, were found in 29.5% of men and 20.9% of women with metabolic risk factors for MASLD (*N* = 2,397). These FIB-4 subgroups were characterized by a significantly higher percentage of subjects aged 80. years and those with heart failure, impaired renal function, and hyperuricemia, but considerably lower percentages of subjects with obesity, hypercholesterolemia, hypertriglyceridemia, and increased HOMA-IR values. In addition, the subgroup of men with higher FIB-4 values was characterized by a significantly lower percentage of diabetics and the subset of women with a lower rate of visceral obesity.

**Figure 2:**
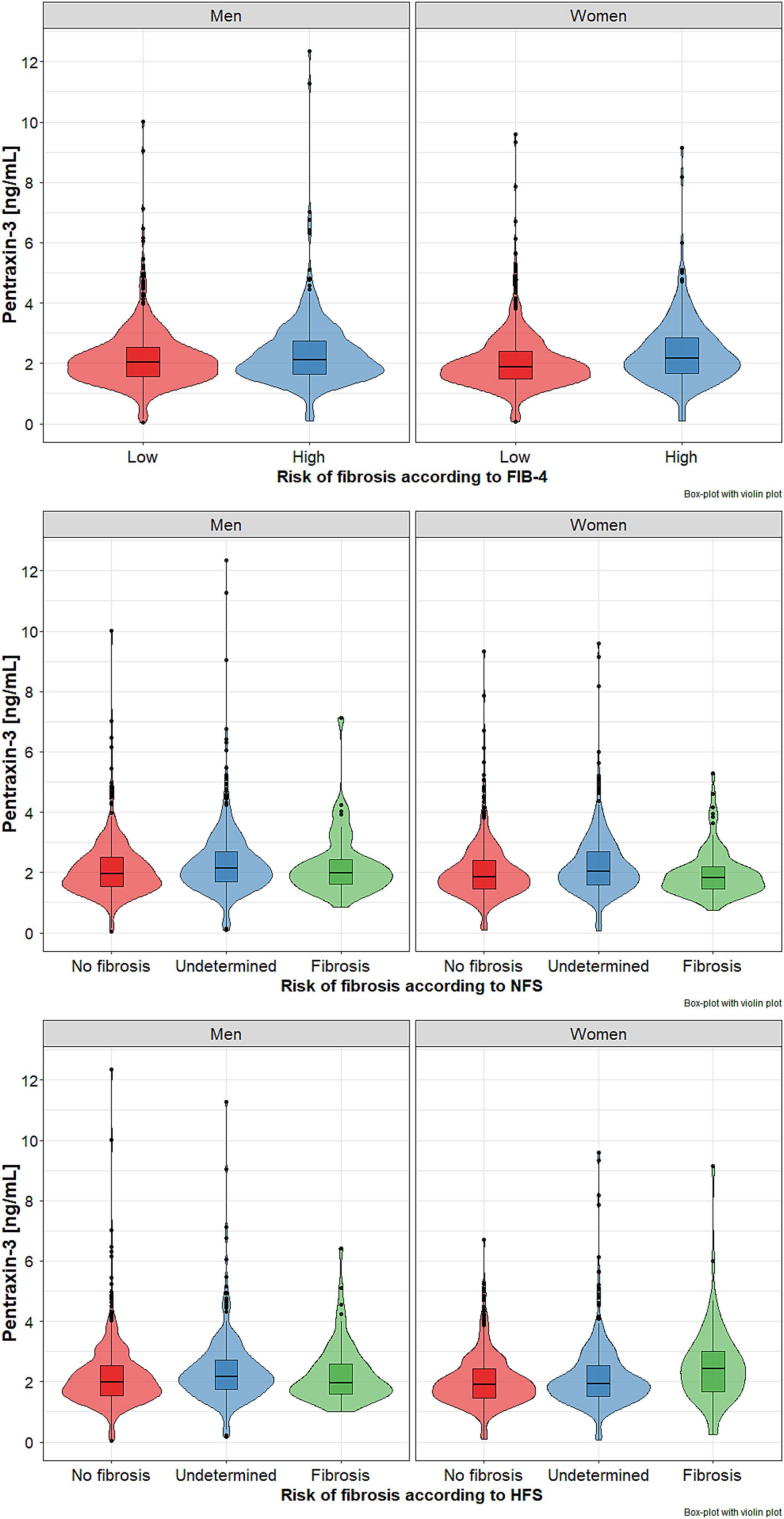
Pentraxin 3 (PTX-3) levels in subgroups of older men and women with metabolic dysfunction-associated steatohepatitis (MASH) with different risk of liver fibrosis based on fibrosis index FIB-4 - (upper panel), NFSs - NAFLD fibrosis scores (middle panel), and HFS - Hepamet fibrosis score (lower panel). Data presented as median (horizontal line) with lower and upper quartiles (boxes). Points represent outliers, while violin plots show the data distribution. Data points that are located outside the fences (“whiskers”) of the boxplot are the so-called outliers. They are defined as points outside 1.5 times the interquartile range above the upper quartile and below the lower quartile.

Plasma PTX-3 levels were significantly higher both in men and women with FIB-4 values >2.67 (Table 1, Figure 2 upper panel).

**Table 1:** Study subgroups with liver fibrosis based on FIB-4 characteristics among subjects with metabolic risk factors for MAFLD (N = 2,397).

	Men		Women	
	FIB-4 ≤ 2.67	FIB-4 > 2.67	FIB-4 ≤ 2.67	FIB-4 > 2.67
N (%)	826 (70.5)	345 (29.5)	970 (79.1)	256 (20.9)
Age ≥ 80 years [N;%]	168 (20.3)	174 (50.4)^#^	235 (24.2)	160 (62.5)^#^
Obesity [N;%]	393 (47.6)	136 (39.4)^*^	552 (56.9)	121 (47.3)^**^
Visceral obesity [N;%]	748 (92.2)	305 (90.5)	936 (98.0)	236 (95.6)^*^
Diabetes [N;%]	358 (43.3)	117 (33.9)^**^	294 (30.3)	87 (34.0)
Hypertension [N;%]	697 (84.4)	301 (87.2)	847 (87.4)	229 (89.5)
Coronary artery disease [N;%]	108 (13.1)	56 (16.2)	73 (7.5)	39 (15.2)^#^
Past stroke [N;%]	86 (10.4)	38 (11.2)	78 (8.1)	20 (7.8)
Heart failure [N;%]	182 (23.0)	111 (34.4)^#^	141 (15.3)	63 (26.6)^#^
Hypercholesterolemia [N;%]	612 (74.1)	250 (72.5)	803 (82.8)	192 (75.0)^**^
Hypertriglyceridemia [N;%]	308 (37.3)	81 (23.5)^#^	387 (39.9)	74 (28.9)^**^
Impaired fasting glucose [N;%]	518 (62.7)	199 (57.7)	473 (48.8)	118 (46.1)
HOMA-IR ≥ 2.0 [N;%]	529 (64.0)	192 (55.6)^**^	595 (61.3)	125 (48.8)^#^
hs-CRP > 3 [N;%]	306 (37.0)	116 (33.6)	405 (41.7)	92 (35.9)
Hyperuricemia [N;%]	256 (31.0)	135 (39.1)^**^	319 (32.9)	97 (37.9)
eGFR <45 mL/min/1.73m^2^ [N;%]	34 (4.1)	33 (9.6)^#^	52 (5.4)	43 (16.8)^#^
Pentraxin 3 [ng/mL]	2.03 (1.57–2.53)	2.13^*^ (1.65–2.74)	1.88 (1.45–2.41)	2.16^#^ (1.68–2.86)

CRP, C-reactive protein; eGFR, estimated glomerular filtration rate; HOMA-IR, homeostatic model assessment of insulin resistance. ^*^p < 0.05. ^**^p < 0.01, ^#^*p* < 0.001, comparison between higher and lower values of FIB-4.

### 3.2 NFS

Based on NFS stratification, no significant risk of fibrosis was found in 3.9% of men and 7.6% of women, while a significant risk of advanced fibrosis in 50.4% of men and 41.3% of women, respectively. Both gender subgroups with considerable risk of advanced fibrosis were characterized by significantly higher percentages of individuals aged 80 years and older, as well as those with obesity, impaired fasting glucose, diabetes, heart failure, hyperuricemia, and, among women only, impaired renal function (Table 2). Plasma PTX-3 levels were significantly higher in both women and men with advanced risk of fibrosis (*p* < 0.001) (Figure 2, middle panel).

**Table 2:** Study subgroups with metabolic risk factors of MAFLD stratified according to liver fibrosis based on the NFS system (*N* = 2,397).

	Men				Women			
NFSs	No significant fibrosis	Undetermined	Advanced fibrosis	*p*	No significant fibrosis	Undetermined	Advanced fibrosis	*p*
N (%)	46 (3.9)	535 (45.7)	590 (50.4)		93 (7.6)	627 (51.1)	506 (41.3)	
Age ≥ 80 years [N;%]	4 (8.7)	80 (14.9)	258 (43.7)^#^	< 0.001	3 (3.2)	128 (20.4)	264 (52.2)^#^	< 0.001
Obesity [N;%]	8 (17.4)	211 (39.4)	310 (52.5)^#^	< 0.001	27 (29.0)	323 (51.5)	323 (63.8)^#^	< 0.001
Visceral obesity [N;%]	39 (86.7)	481 (92.0)	533 (91.9)	0.45	89 (95.7)	602 (97.9)	481 (97.4)	0.44
Dibetes [N;%]	10 (21.7)	171 (32.0)	294 (49.8)^#^	< 0.001	12 (12.9)	154 (24.6)	215 (42.5)^#^	< 0.001
Hypertension [N;%]	43 (93.5)	448 (83.7)	507 (85.9)	0.16	81 (87.1)	538 (85.9)	457 (90.3)	0.08
Coronary artery disease [N;%]	5 (10.9)	66 (12.3)	93 (15.8)	0.21	4 (4.3)	47 (7.5)	61 (12.1)^*^	< 0.01
Stroke [N;%]	3 (6.5)	52 (9.7)	69 (11.8)	0.35	3 (3.2)	46 (7.4)	49 (9.8)	0.07
Heart failure [N;%]	9 (20.9)	94 (18.4)	190 (34.0)	< 0.001	7 (7.9)	85 (14.2)	112 (23.8)^#^	< 0.001
Hypercholesterolemia [N;%]	35 (76.1)	414 (77.4)	413 (70.0)	< 0.05	84 (90.3)	535 (85.3)	376 (74.3)^#^	< 0.001
Hypertriglyceridemia [N;%]	24 (52.2)	208 (38.9)	157 (26.6)^#^	< 0.001	44 (47.3)	250 (39.9)	167 (33.0)^#^	< 0.01
Impaired fasting glucose [N;%]	16 (34.8)	283 (52.9)	418 (70.8)^#^	< 0.001	18 (19.3)	255 (40.7)	318 (62.8)^#^	< 0.001
HOMA-IR ≥ 2.0 [N;%]	26 (56.5)	304 (56.8)	391 (66.3)	< 0.01	52 (55.9)	350 (55.8)	318 (62.8)	< 0.05
Hyperuricemia [N;%]	11 (23.9)	148 (27.7)	232 (39.3)^*^	< 0.001	21 (22.6)	182 (29.0)	213 (42.1)^#^	< 0.001
eGFR <45 mL/min/1.73m^2^ [N;%]	2 (4.3)	17 (3.2)	48 (8.1)	< 0.01	0	18 (2.9)	77 (15.2)^#^	< 0.001
Pentraxin 3 [ng/mL]	1.99 (1.62–2.46)	1.96 (1.53–2.50)	2.13^#^ (1.69–2.70)	< 0.05	1.82 (1.46–2.20)	1.86 (1.46–2.41)	2.05^#^ (1.58–2.68)	< 0.01

eGFR – estimated glomerular filtration rate, HOMA-IR - homeostatic model assessment of insulin resistance. *p < 0.05; #*p* < 0.001, comparison between no significant fibrosis and advanced fibrosis in corresponding subgroups of men and women.

### 3.3 HFS

There were much lower rates of subjects at risk of advanced fibrosis based on the HFS System compared to FIB-4 and NFS stratifications. Only 6.2% of men and 6.1% of women met the criteria of advanced fibrosis. Both subgroups at risk of considerable fibrosis were characterized by significantly higher percentages of subjects aged 80 years and over, those with impaired fasting glucose, diabetes, heart failure, hyperuricemia, and impaired renal function. The frequency of obesity was significantly higher in men with the undetermined group, while visceral obesity was in women with no advanced fibrosis group. The highest PTX-3 levels were noted in men with undermined risk and women with a significant risk of fibrosis; however, the difference was statistically significant only between undetermined and no advanced fibrosis subgroups (Table 3, Figure 2 – lower panel).

**Table 3:** Study subgroups with metabolic risk factors of MAFLD stratified according to liver fibrosis based on the HFS system (*N* = 2,397).

	Men				Women			
	No significant fibrosis	Undetermined	Advanced fibrosis	p	No significant fibrosis	Undetermined	Advanced fibrosis	p
N (%)	733 (62.6)	365 (31.2)	73 (6.2)		611 (49.8)	540 (44.1)	75 (6.1)	
Age ≥ 80 years [N;%]	177 (24.1)	135 (37.0)^#^	30 (41.1)^**^	< 0.001	163 (26.7)	199 (36.8)^#^	33 (44.0)^**^	< 0.001
Obesity [N;%]	308 (42.0)	186 (51.0)^**^	35 (47.9)	< 0.05	320 (52.4)	312 (57.8)	41 (54.7)	0.18
Visceral obesity [N;%]	672 (92.7)	321 (91.2)	60 (84.5)^*^	0.06	590 (98.3)	516 (97.4)	66 (91.7)^**^	< 0.01
Dibetes [N;%]	160 (21.8)	248 (67.9)^#^	67 (91.8)^#^	< 0.001	26 (4.3)	285 (52.8)^#^	70 (93.3)^#^	< 0.001
Hypertension [N;%]	619 (84.4)	319 (87.4)	60 (82.2)	0.32	530 (86.7)	481 (89.2)	65 (86.7)	0.41
Coronary artery disease [N;%]	92 (37.2)	57 (35.8)	15 (45.4)	0.58	49 (40.8)	52 (36.6)	11 (35.5)	0.74
Stroke [N;%]	74 (10.1)	39 (10.8)	11 (15.1)	0.42	51 (8.4)	39 (7.3)	8 (10.7)	0.56
Heart failure [N;%]	153 (21.8)	110 (32.3)^#^	30 (41.7)^#^	< 0.001	77 (13.2)	110 (21.7)^#^	17 (24.3)^*^	< 0.001
Hypercholesterolemia [N;%]	554 (75.6)	259 (71.0)	49 (67.1)	0.11	530 (86.7)	411 (76.1)^#^	54 (72.0)^**^	< 0.001
Hypertriglyceridemia [N;%]	264 (36.0)	102 (28.0)^**^	23 (31.5)	< 0.05	236 (38.6)	195 (36.1)	30 (40.0)	0.62
Impaired fasting glucose [N;%]	389 (53.1)	269 (73.7)^#^	59 (80.8)^#^	< 0.001	196 (32.1)	335 (62.0)^#^	60 (80.0)^#^	< 0.001
HOMA-IR ≥ 2.0 [N;%]	397 (54.2)	267 (73.1)^#^	57 (78.1)^#^	< 0.001	260 (42.6)	404 (74.8)^#^	56 (74.7)^#^	< 0.001
Hyperuricemia [N;%]	225 (30.7)	138 (37.8)^*^	28 (38.4)	< 0.05	180 (29.5)	199 (36.8)^**^	37 (49.3)^#^	< 0.001
eGFR <45 mL/min/1.73m^2^ [N;%]	33 (4.5)	30 (8.2)^*^	4 (5.5)	< 0.05	30 (4.9)	55 (10.2)^#^	10 (13.3)^**^	< 0.001
Pentraxin 3 [ng/mL]	1.98 (1.54–2.52)	2.17^#^ (1.76–2.71)	1.95 (1.60–2.59)	< 0.01	1.89 (1.47–2.42)	1.94^#^ (1.52–2.53)	2.42 (1.65–3.04)	< 0.01

eGFR, estimated glomerular filtration rate; HOMA-IR, homeostatic model assessment of insulin resistance. ^*^p < 0.05; ^#^*p* < 0.001 – comparison groups to group with no advanced fibrosis.

### 3.4 Cutoff PTX-3 values

The empirical cutoff points for PTX-3 levels as a potential marker of liver fibrosis were assessed separately for women and men (Table 4).

**Table 4:** Results of the ROC analysis for all three fibrosis markers, separately for men and women.

	FIB-4 fibrosis		NFSs		HFS	
	Men	Women	Men	Women	Men	Women
PTX-3 cutoff [ng/mL]	≥ 2.39	≥ 1.96	≥ 2.48	≥ 2.25	≥ 1.43	≥ 2.30
AUC	0.549 (0.513–0.585)	0.608 (0.568–0.647)	0.537 (0.451–0.624)	0.596 (0.536–0.657)	0.529 (0.460–0.597)	0.643 (0.572–0.714)
p	< 0.01	< 0.001	0.40	< 0.01	0.41	< 0.001
Sensitivity [%]	39.4 (34.2–44.8)	61.7 (55.5–67.7)	33.7 (29.9–37.7)	39.1 (34.8–43.5)	91.8 (83.0–96.9)	54.7 (42.7–66.2)
Specificity [%]	69.6 (66.3–72.7)	56.1 (52.9–59.2)	76.1 (61.2–87.4)	79.6 (70.0–87.2)	19.0 (7.9–12.7)	73.0 (69.3–76.5)
PPV [%]	35.1 (30.4–40.1)	27.1 (23.5–30.9)	94.8 (90.8–97.4)	91.2 (86.7–94.6)	10.1 (7.9–12.7)	19.9 (14.7–26.0)
NPV [%]	73.3 (70.1–76.4)	84.7 (81.7–87.4)	8.2 (5.8–11.2)	19.4 (15.5–23.7)	95.9 (91.2–98.5)	92.3 (90.2–95.0)
ACC	60.7 (57.8–63.5)	57.3 (54.4–60.0)	36.8 (33.0–40.7)	45.4 (41.4–49.5)	25.6 (22.6–28.7)	71.0 (67.4–74.4)
Diagnostic OR	1.49 (1.15–1.94)	2.06 (1.55–2.73)	1.62 (0.80–3.26)	2.50 (1.47–4.27)	2.61 (1.11–6.15)	3.26 (2.00–5.31)

AUC, area under curve; PPV, positive predictive values; NPV, negative predictive value; ACC, accuracy; OR, odds ratio; brackets represent 95% confidence intervals.

The cutoff points for PTX-3 levels based on all significant ROC curve analyses, as a marker of liver fibrosis, ranged from 1.96 to 2.30 ng/mL (an AUC ranging between 0.596 and 0.643, sensitivity from 39.1 to 61.7%, and specificity from 56.1 to 79.6%) in women. In men, a significant cutoff point was established only for FIB-4 (an AUC of 0.549, sensitivity of 39.4%, and specificity of 69.6%). The accuracy was poor (Table 4).

## 4 Discussion

To the best of our knowledge, this is the first study that assessed plasma PTX-3 levels as a marker of liver fibrosis in the older adult population. Obesity is the main factor in the development of MASLD (1). It has been suggested that PTX-3 is a link between obesity, inflammation, and metabolic and cardiovascular complications of obesity (23). In middle-aged subjects, plasma PTX-3 levels were shown to increase alongside BMI and waist circumference (24). However, in our study, there was a significantly lower percentage of old-age subjects with obesity in both women and men with liver fibrosis, assessed based on FIB-4 scores and HFSs. In addition, a significantly lower rate of subjects with visceral obesity was found among men with a high risk of liver fibrosis assessed based on FIB-4 scores and in women on the HFSs. These differences are difficult to explain, but hypotheses can be put forward. First, there are no well-established criteria for diagnosing obesity according to the WHO criteria and visceral obesity in long-lived adults. The increasing with age incidence of sarcopenic obesity should be noted. Moreover, it remains unknown whether waist circumference is a sensitive and adequate parameter of visceral fat accumulation in older people. The waist circumference to height ratio is suggested as a better diagnostic parameter of visceral obesity in older people (25). Second, among people with liver fibrosis, there is a much higher percentage of people with heart failure and impaired kidney function, which could have contributed to the development of malnutrition, resulting in a reduction in their body weight. Nevertheless, visceral fat accumulation plays a key role in MASLD development in young male adults, even non-obese (26). It should be noted that the duration of MASLD and other diseases was not assessed in our study, which could be important for analyzing body weight changes and their relationship with the diseases occurring in the subjects. Recently, attention has been drawn to increased cardiovascular risk associated with MAFLD occurrence (27, 28). In turn, arterial hypertension and coronary heart disease are the main causes of the development of heart failure (29–31). Moreover, the association between MAFLD and chronic kidney disease (CKD) was observed (28). Thus, MASLD is a link between obesity, cardiovascular diseases, heart failure, and CKD. On the other hand, age and multi-morbidity are associated with the risk of developing malnutrition, therefore, searching for relationships between obesity and visceral obesity and their known complications becomes very difficult. Explanation of the sequence of events and their relationship with body weight would require large prospective long-term observation.

Plasma PTX-3 levels in our study were significantly higher in subgroups with a high risk of advanced liver fibrosis assessed based on the FIB-4 scores and NFSs. Notwithstanding, the highest PTX-3 levels were noted in men with undetermined risk and women with a significant risk of fibrosis according to the HFSs; however, the difference was statistically significant only between undetermined and no advanced fibrosis subgroups. Of interest, when liver fibrosis was assessed based on the NFSs, both sex subgroups with advanced fibrosis were characterized by significantly higher percentages of subjects aged 80 years and older, those with obesity, visceral obesity, diabetes, hypertension, heart failure, impaired fasting glucose, insulin resistance, and impaired kidney function. Similarly, when liver fibrosis was assessed based on the HFSs, both sex subgroups with advanced fibrosis were characterized by significantly higher percentages of subjects aged 80 years and older, those with impaired fasting glucose, diabetes, heart failure, hyperuricemia, and impaired renal function. These results suggest that both the NFSs and HFSs are more adequate than FIB-4 for liver fibrosis assessment in older adults. Since it does not cause as much controversy as the results obtained in evaluating fibrosis risk based on FIB-4. There is a lack of data regarding the usefulness of individual non-invasive tests, including FIB-4 and HFS, for assessing liver fibrosis in older adults. Therefore, studies are needed to determine which of the markers best correlates with fibrosis assessed by MRI or liver biopsy in old and very old adults. However, it should be noted that it has been demonstrated that HFS identified patients with advanced fibrosis with higher levels of accuracy than the NAFLD Fibrosis Score and Fibrosis-4 scores (11).

In this context, it is worth searching for plasma PTX-3 concentrations as a potential biomarker that could replace non-invasive liver fibrosis tests. In our study, plasma PTX-3 levels were significantly higher in both women and men with liver fibrosis assessed based on FIB-4. However, when the risk of liver fibrosis was calculated according to NFSs, plasma PTX-3 levels were significantly higher in both men and women with advanced fibrosis. The assessment of liver fibrosis based on the HFS showed the highest PTX-3 levels in men with undermined risk and women with a significant risk of advanced fibrosis. However, the difference was statistically significant only between undetermined and no advanced fibrosis subgroups. Our results correspond with those obtained in middle-aged subjects, where plasma PTX-3 levels were significantly higher in stage F3–F4 than F0–F2 NAFLD cases, and plasma PTX-3 concentrations correlated with stages of liver fibrosis regardless of sex (15). These results raised a question concerning the clinical usefulness of PTX-3 assessment as a biomarker of liver fibrosis in older adults.

We have tried to establish the empirical cutoff point for PTX-3 level as a marker of liver fibrosis assessed based on FIB-4, NFS, and HFS system values, separately for men and women. Unfortunately, all cutoff points showed low specificity and accuracy, precluding their use in clinical practice. Contrary to results obtained in the middle-aged population (sensitivity 91.1% and specificity 71.4% for PTX-3 cutoff of 2.45 ng/mL) (14) and adolescents with obesity (sensitivity 89.0% and specificity 86.0% for PTX-3 cutoff of 3.03 ng/mL) (16). The observed discrepancies are probably the consequences of aging.

As was mentioned above, numerous factors could affect the content of adipose tissue and, indirectly, the concentration of PTX-3. Moreover, the influence of chronically used medications by our study participants on PTX-3 concentrations cannot be ruled out. Another factor reducing the cutoff point value and sensitivity and specificity of PTX-3 as a marker of liver fibrosis may be the fatty liver and NASH duration. This hypothesis is supported by our previous study that showed that the increase of plasma TNF-*α* level is an early event in abdominal fat accumulation, and further fat mass gain does not enhance circulating TNF-α levels (32). In addition, it was reported that adipocytes induce PTX-3 synthesis by secreting various inflammatory cytokines (33). Thus, biomarkers that seem useful in young and middle-aged people may have limited utility in old and very old adults, as indicated by the results of our study.

The study’s main limitation is the lack of an assessment of liver fibrosis based on elastography, liver biopsy, or nuclear magnetic resonance imaging. The second one is the assessment of visceral obesity based on waist circumference and not on the visceral fat area assessment using computed tomography or magnetic resonance. However, implementing such methods in a population-based study is complicated and expensive. Other limitations included the inability to assess the impact of medication on plasma PTX-3 concentrations due to the lack of identification of drugs affecting plasma PTX-3 levels and the lack of assessment of changes over time. The limitations also include the lack of the evaluation of complementary markers and the use of imaging techniques to evaluate liver fibrosis. Thus, further studies are required to elucidate the role of PTX-3 as a marker of liver fibrosis in older adults.

The strength of the study is its large sample size and the representativeness of participants within the Polish population. Moreover, the usefulness of PTX-3 as a biomarker of liver fibrosis in the older population has not been assessed so far.

## 5 Conclusion

Our study suggests that plasma PTX-3 levels are not sensitive enough to be used as a non-specific marker of liver fibrosis in older adults.

## Data Availability

The raw data supporting the conclusions of this article will be made available by the authors, without undue reservation.
